# Climate change has different predicted effects on the range shifts of two hybridizing ambush bug (*Phymata*, Family *Reduviidae*, Order Hemiptera) species

**DOI:** 10.1002/ece3.6820

**Published:** 2020-10-15

**Authors:** Vicki Mengyuan Zhang, David Punzalan, Locke Rowe

**Affiliations:** ^1^ Department of Ecology and Evolutionary Biology University of Toronto Toronto ON Canada; ^2^ Department of Biology University of Toronto Mississauga ON Canada; ^3^ Department of Biology University of Victoria Victoria BC Canada

**Keywords:** abiotic, bioclimatic variables, climate change, Maxent, range shifts, species distribution modeling

## Abstract

**Aim:**

A universal attribute of species is that their distributions are limited by numerous factors that may be difficult to quantify. Furthermore, climate change‐induced range shifts have been reported in many taxa, and understanding the implications of these shifts remains a priority and a challenge. Here, we use Maxent to predict current suitable habitat and to project future distributions of two closely related, parapatrically distributed *Phymata* species in light of anthropogenic climate change.

**Location:**

North America.

**Taxon:**

*Phymata americana* Melin 1930 and *Phymata pennsylvanica* Handlirsch 1897, Family: *Reduviidae*, Order: Hemiptera.

**Methods:**

We used the maximum entropy modeling software Maxent to identify environmental variables maintaining the distribution of two *Phymata* species, *Phymata americana* and *Phymata pennsylvanica*. Species occurrence data were collected from museum databases, and environmental data were collected from WorldClim. Once we gathered distribution maps for both species, we created binary suitability maps of current distributions. To predict future distributions in 2050 and 2070, the same environmental variables were used, this time under four different representative concentration pathways: RCP2.6, RCP4.5, RCP6.0, and RCP8.5; as well, binary suitability maps of future distributions were also created. To visualize potential future hybridization, the degree of overlap between the two *Phymata* species was calculated.

**Results:**

The strongest predictor to *P. americana* ranges was the mean temperature of the warmest quarter, while precipitation of the driest month and mean temperature of the warmest quarter were strong predictors of *P. pennsylvanica* ranges. Future ranges for *P. americana* are predicted to increase northwestward at higher CO_2_ concentrations. Suitable ranges for *P. pennsylvanica* are predicted to decrease with slight fluctuations around range edges. There is an increase in overlapping ranges of the two species in all future predictions.

**Main conclusions:**

These evidences for different environmental requirements for *P. americana* and *P. pennsylvanica* account for their distinct ranges. Because these species are ecologically similar and can hybridize, climate change has potentially important eco‐evolutionary ramifications. Overall, our results are consistent with effects of climate change that are highly variable across species, geographic regions, and over time.

## Graphical abstract

A universal attribute of species is that their distributions are limited by numerous factors that may be difficult to quantify, but the identification of these factors is a priority given climate change‐induced range shifts that may have ecological and evolutionary implications. We use Maxent to predict current suitable habitat and to project future distributions of two closely related, parapatrically distributed *Phymata* species in light of anthropogenic climate change. We found evidence for different environmental requirements for *P. americana* and *P. pennsylvanica*, accounting for their distinct ranges predicted current and future ranges, and argue that climate change has potentially important eco‐evolutionary ramifications on both species.

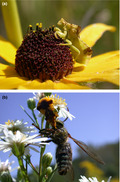

## 1 INTRODUCTION

A universal attribute of all species is that their geographic distributions are limited. The abiotic and biotic factors that jointly determine this distribution are expected to be numerous, posing a serious empirical challenge to their identification and quantification. One approach is to employ species distribution models (SDMs), which attempt to explain species presence data with a large set of predictor variables (Elith & Leathwick, 2009). Although the approach is generally limited to the use of environmental variables (or their proxies) as predictors of suitable habitat, SDM have provided new insights into species requirements that are akin to the “fundamental niche” (Hutchinson, 1957) by incorporating constraints set by biotic interactions (Leach et al., 2016).

Geographic distributions are dynamic and dependent upon changing environmental conditions. Species also vary in their sensitivity to shifting environmental conditions and will respond differently to the same changes (Hickling et al., 2006; Malcolm et al., 2002), including the possibility of failure to track new conditions altogether (Loarie et al., 2009). While there is growing evidence of climate change‐induced range shifts in many taxa, predicting its ecological and evolutionary implications remains a central challenge (Parmesan, 2006). For example, climatic variation is undoubtedly linked to natural changes in community composition over geological timescales, and there is growing evidence of rapid changes in climate being linked to the invasion and expansion of alien species (Bellard et al., 2013; Guo et al., 2012). Changing climatic conditions has also led to more frequent contact between historically separate species, and this can result in hybridization (Vallejo‐Marín & Hiscock, 2016) and, in some cases, species collapse (Njiru et al., 2010). For these reasons, the responses of hybridizing species to environmental change have been touted as a particularly important “window” on climate change (Taylor et al., 2015).

**FIGURE 1:**
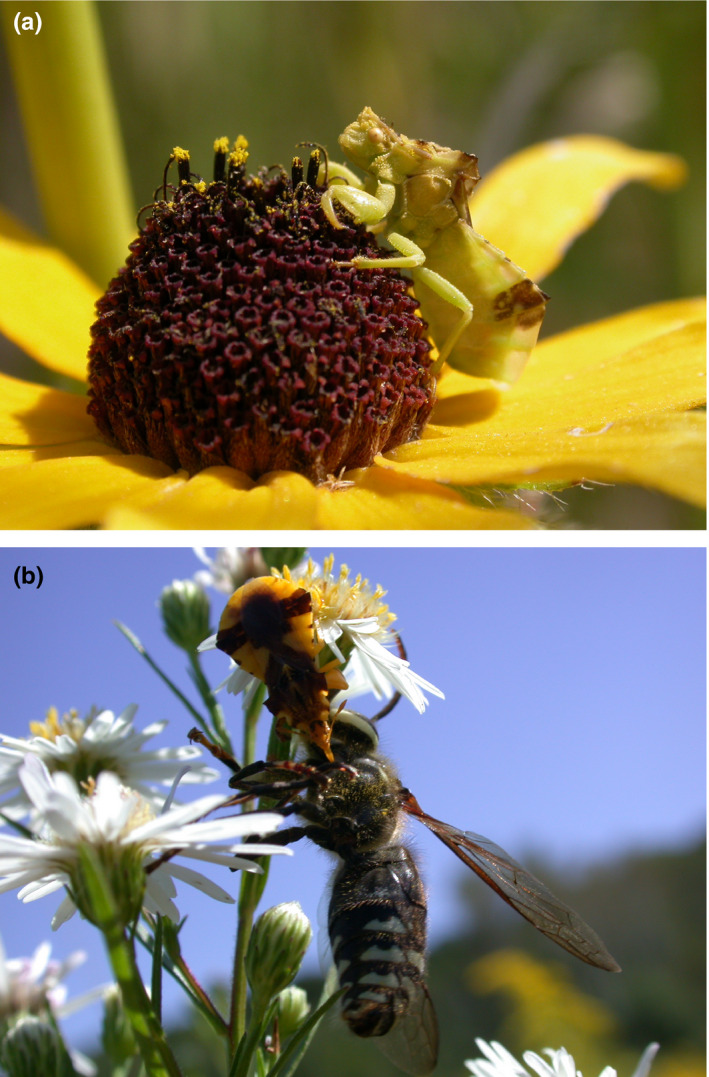
Photographs of the two organisms in the present study: (a) *P. americana* waiting for prey on a Black‐eyed Susan (*Rudbeckia hirta*); (b) *P. pennsylvanica* consuming a bald‐faced hornet (*Dolichovespula maculate*). Images by David Punzalan

In the present paper, we evaluate potential range shifts in a parapatric pair of insect species. *Phymata* *americana* Melin 1930 and *P. pennsylvanica* Handlirsch 1897 (Figure 1) are two of the most common North American species in the genus (Family: *Reduviidae*, Order: Hemiptera), with the former more northerly in distribution, extending west across the American Midwest and Canadian prairies, and the latter mostly concentrated in the northeastern United States. Hybridization in wild populations has been suspected or inferred in overlapping regions of their ranges (Punzalan & Rowe, 2017; Swanson, 2013); consistent with this, current molecular phylogenetic data fail to distinguish between the two (Masonick et al., 2017; Masonick & Weirauch, 2020), despite substantial morphological divergence (Punzalan & Rowe, 2017). Both species are generalist predators occurring in temperate habitats, where they utilize a wide range of plant species as hunting sites (Balduf, 1939, 1941; Yong, 2005), suggesting considerable niche overlap. In at least one of the species, climatic variables (e.g., environmental temperature) play a key role in thermoregulation, which is linked to mating activity (Punzalan et al., 2008a). Thermoregulatory abilities have been linked to melanic traits, and within‐ and between‐species latitudinal variation in these traits (Punzalan & Rowe, 2015) indirectly supports the importance of climatic variables in the ecology of *Phymata*. Although there is evidence that their ecological requirements are consequential to their life histories, there is a shortage of knowledge regarding their ecology. Thus, there is value in understanding their habitat requirements and predicting their current and future ranges.

We used biogeographic climate data and SDM to characterize the current and recent historical range of *P. americana* and *P. pennsylvanica* and forecast future distributions under several scenarios of anthropogenic climate change. We hypothesized that different sets of candidate abiotic factors limit the respective ranges of the two species, resulting in their current distributions. Given anthropogenic climate warming, we also hypothesized that their future overlapping ranges would increase.

## 2 MATERIALS AND METHODS

Ambush bug distribution data were compiled from specimens examined by one of the authors (DP) at the American Museum of Natural History (AMNH), Carnegie Museums of Pittsburgh, Canadian National Collection of Insects, Arachnids, and Nematodes, Royal Ontario Museum, Smithsonian National Museum, University of Guelph Insect Collection, and the University of Michigan Museum of Zoology. Identifications considered questionable by DP were excluded from subsequent analyses. These data were supplemented with information available from museum databases provided by the Spencer Entomological Collection, at the Beaty Biodiversity Museum (https://www.zoology.ubc.ca/entomology/) and the Plant Bug Inventory maintained by the AMNH (http://research.amnh.org/pbi/). We also gathered data from two citizen science websites, iNaturalist.org and BugGuide.net. Data collected from iNaturalist.org were included if the species identity was verified by Paul Masonick (UC Riverside), an authority on *Phymata* systematics and curator of the iNaturalist project “Uncovering the ambush bugs” (https://www.inaturalist.org/projects/uncovering-the-ambush-bugs). Data collected from BugGuide.net require secondary identification verification before publishing on the *Phymata* webpage and were assumed to be accurate.

**FIGURE 2:**
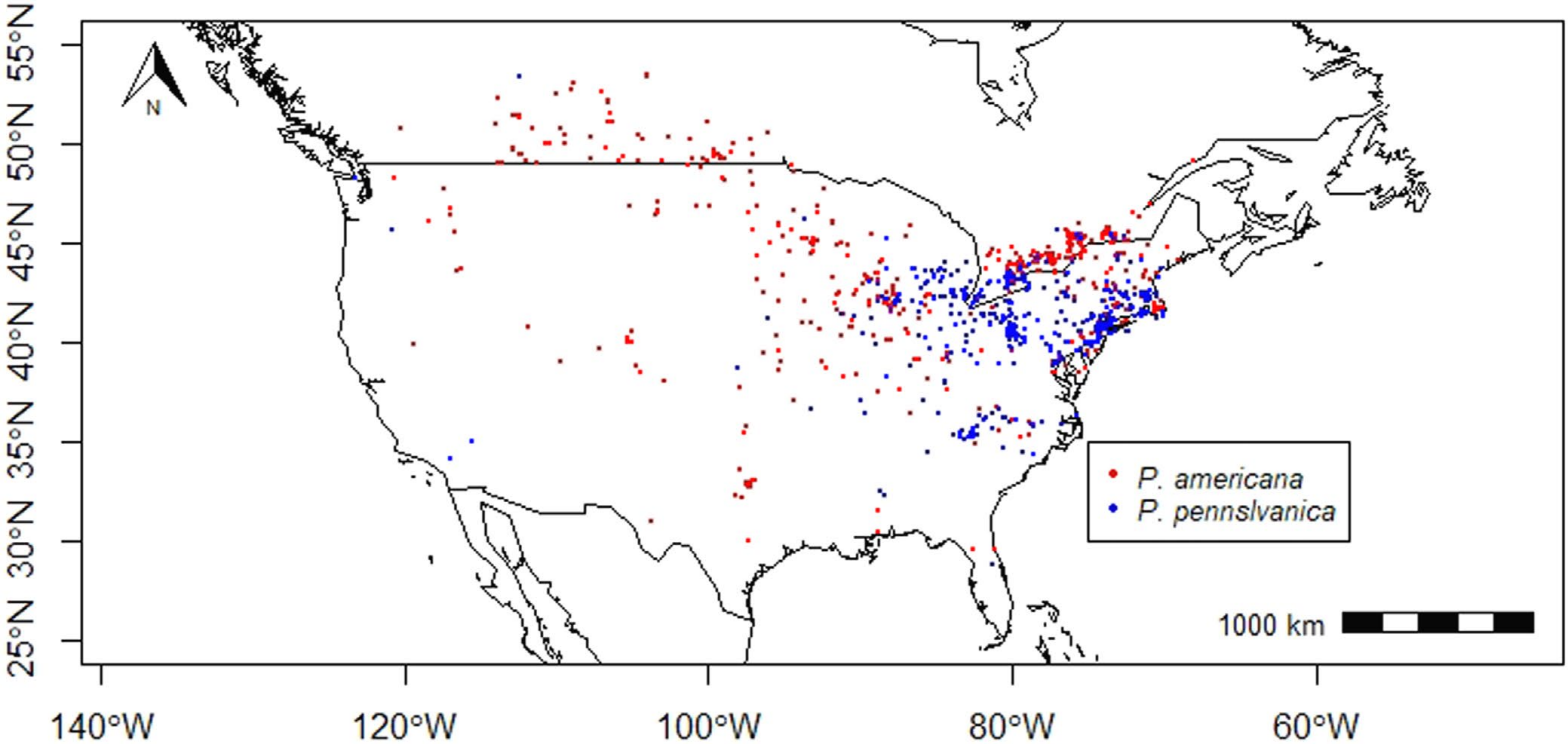
Locations of *P. americana* sightings in red (1,075 observations) and *P. pennsylvanica* sightings in blue (970 observations); this includes both museum and citizen science data and spans a time frame from 1864 to 2018

For accessions lacking latitude and longitude data, we supplemented these data manually using Google Earth Pro version 7.3.1 (Google Earth, 2018). Given that bug sightings occurred at a specific point, but its accuracy was not reflected on Google Earth, coordinates were rounded to include degree and hour only (i.e., minutes and seconds were not used). This is because locality data were only accurate down to the city level and, in some cases, were accurate down to the location of the field station, research station, or building. For the present purposes, degrees and hours should be sufficient. The localities of all sightings are mapped in Figure 2.

Although museum and citizen science databases go to lengths to confirm species identifications, the possibility of misidentifying individuals is inevitable, as is heterogeneity in sampling methods and reporting. To mitigate the effects of such errors, the data sets were inspected, and we removed any data points that were outside of the range of North America that were a result of errors in the data entry of latitudes or longitudes, as well as any species identifications that were unreliable (i.e., indicated as uncertain by the collector/photographer). There were a total of 1,075 observations of *P. americana* and 970 observations of *P. pennsylvanica*.

Locality data were also divided into two subsets, according to “historical,” referring to sightings before 1970, and “current” referring to sightings from 1970 to present. Preliminary inspection of the “current” and “historical” distributions of each species suggests that median latitudes of *P. americana* have decreased, indicating a southward shift (Mann–Whitney *U* = 109,360, *n* = 1,075, *p*‐value = .00019), while *P. pennsylvanica* median latitudes have increased, indicating a northward shift (*U* = 127,290, *n* = 970, *p*‐value = .00076). This was consistent with our suspicion that there are opposing changes in the ranges of both species, and motivated the current study. Additionally, visual comparisons of the historical and current distributions suggested an increase in the overlap of *P. americana* and *P. pennsylvanica* distributions (Figure S1.1 in Appendix SS1). For subsequent analyses, we restricted the data from 1970 to 2000 (see Table S1.1, Appendix S1), and we refer to this as the current distribution. In this restricted data set, there were 226 observations: 104 (62 unique) observations of *P. americana* and 122 (66 unique) observations of *P. pennsylvanica*. This was done in order to prevent temporal mismatch with the bioclimatic data which contained environmental data from 1970 to 2000.

We obtained climate data in raster form from the WorldClim database (Fick & Hijmans, 2017), which covered all global land areas except for Antarctica. The grid data were in 2.5 arcminutes (approximately 4.5 square kilometers at the equator). The updated (2.0) version of WorldClim's current environmental data was used to investigate the influence of environmental variables on ambush bug distributions. This environmental data ranged from 1970 to 2000 and included 19 bioclimatic variables (Table S1.2, Appendix S1) derived from monthly temperature and precipitation measurements. To detect predictor collinearity, we used a variance inflation factor (VIF) from the “usdm” package (Naimi et al., 2014) in R version 4.0.2 (R Core Team). We excluded variables with that exceeded a VIF correlation threshold of 10 (Miles, 2014), and then, we verified that the remaining variables are presumed to be more relevant to *Phymata* ecology (Brandt et al., 2017), and contributed more than 0% to the models based on numerous Maxent runs (see below). This resulted in 8 bioclimatic variables for *P. americana* and *P. pennsylvanica* (Table S1.2, Appendix S1): Mean Diurnal Range (BIO2), Temperature Annual Range (BIO7), Mean Temperature of Wettest Quarter (BIO8), Mean Temperature of Warmest Quarter (BIO10), Precipitation of Driest Month (BIO14), Precipitation Seasonality (BIO15), Precipitation of Warmest Quarter (BIO18), and Precipitation of Coldest Quarter (BIO19).

To map future distributions, data from future climate variables were collected from WorldClim's original (1.4) version (as there is currently no updated 2.0 version), using the same 8 bioclimatic variables. We used the global circulation model Community Climate System Model (CCSM) version 4 as future climatic data. Climate variables were projected into 2050 and 2070, with four representative concentration pathway (RCP) trajectories representing four possible climate change scenarios dependent on atmospheric greenhouse gas concentration. The best‐case scenario for atmospheric greenhouse gases is represented by the lowest RCP of 2.6. As RCP increases, the atmospheric greenhouse gas concentration increases as well, up to RCP8.5, the business‐as‐usual scenario. We used maximum entropy modeling of species’ geographic distributions (Maxent version 3.4.1), an approach often favored when restricted to presence data and considered robust even when data are limited. In the absence of information about environmental conditions, we assumed the probability of a species’ occurrence within a grid was 0.5 (the default). When a species was found within a grid for which there is information about environmental conditions, Maxent improves the model using the environmental variables.

We assumed a logistic output format in all runs, which gives an estimate of the probability of ambush bug presence within a grid. We set our parameters to 10 replicates and set the replicated run type to “crossvalidate,” and subsequent analyses were based on the averaged values. We also produced background predictions, response curves, and jacknife plots by checking the relevant boxes. All other Maxent settings were set to default. To evaluate model performance, we calculated the true skill statistic (TSS), which has been demonstrated to be an accurate measure of performance (Allouche et al., 2006). Collinearity shifts when predicting species distributions in future scenarios have been recognized as a source of predictive error in Maxent models (Feng et al., 2015; Júnior & Nóbrega, 2018). We quantified collinearity shift as an assessment of model predictive accuracy by comparing the correlation matrices of current bioclimatic predictor variables and future bioclimatic variables.

We inspected response curves and jacknife plots to evaluate the effect of different continuous environmental variables and to determine the relative importance of these variables. Response curves were generated for each individual predictor, while all other predictors were set at their average. Geographical projections of the models in the form of heat maps were used to visualize the predicted probability of ambush bug occurrence under current and future environmental conditions. Heat maps were then converted to binary presence/absence maps using thresholds using the R package “raster.” The threshold used to create binary maps was “10th percentile training presence logistic threshold.” This threshold was selected as it assumes that 90% of the predicted occurrences will accurately predict the potential range, while 10% of the predicted occurrences may be erroneous. This results in a more conservative threshold and is more commonly used with species distribution data collected over a longer period of time by different observers (Rebelo & Jones, 2010).

Raster math calculations were drawn from methods used to calculate the “suitability status change index” (SSCI), adopted from Ceccarelli and Rabinovich (2015). In order to compare the change in suitable habitat, the future predicted distribution was subtracted from the current predicted distribution. For both *P. americana* and *P. pennsylvanica*, current suitable habitat was classified as “1” and current unsuitable habitat was classified as “0,” while future suitable habitat was classified as “2” and future unsuitable habitat was classified as “0.” The difference between current and future predicted distributions resulted in: “‐1” = suitable habitat will become unsuitable; “0” = unsuitable habitat remains unsuitable; “1” = suitable habitat remains suitable”; and “2” = unsuitable habitat becomes suitable.

We calculated the percent of overlap between *P. americana* and *P. pennsylvanica* projected for 2050 and 2070 to assess changes in potential contact zones. The predicted ranges of *P. americana* were subtracted from the predicted ranges of *P. pennsylvanica*, and the same method was used to calculate the change in suitable habitats. The suitable habitat of *P. pennsylvanica* was reclassified to be “0” for unsuitable habitat, and “2” for suitable habitat, while the current suitable habitat and current unsuitable habitat remained “1” and “0,” respectively, for *P. americana*. Subtracting rasters resulted in: “−1” = suitable habitat for *P. americana* only; “0” = unsuitable habitat for both species; “1” = suitable habitat for both species, indicating potential overlap; and “2” = suitable habitat for *P. pennsylvanica* only. The attribute table for each generated map gives the total number of grids for suitable and unsuitable habitats. Using these ratios, the percent change in future predicted distributions under different RCP trajectories and the degree of overlap between the two species was quantified.

## 3 RESULTS

There were a total of 226 observations within the interval for which climate data were available, 104 for *P. americana* and 122 for *P. pennsylvanica* (Table S1.1, Appendix S1). Using these data, the models produced by the Maxent approach were statistically well supported, as the ratio of true positives (i.e., sensitivity) to false positives (i.e., 1‐specificity) was maximized.

Collinearity shifts are presented by comparing the correlation matrix of the eight current bioclimatic variables and the average correlation matrix of the same eight bioclimatic variables in all future scenarios (Table S1.3 and Table S1.4, Appendix S1). The largest absolute difference between current and future bioclimatic correlation matrices is a change of 0.13 between BIO7 and BIO10. We regard this small amount of change to indicate that shifts in collinearity are minimal and do not distort the model predictions (Dormann et al., 2013). Model evaluation metrics indicate that the model performed well: for both species’ models, AUC > 0.95 and TSS > 0.7 (Table S2.1, Appendix S2).

**FIGURE 3:**
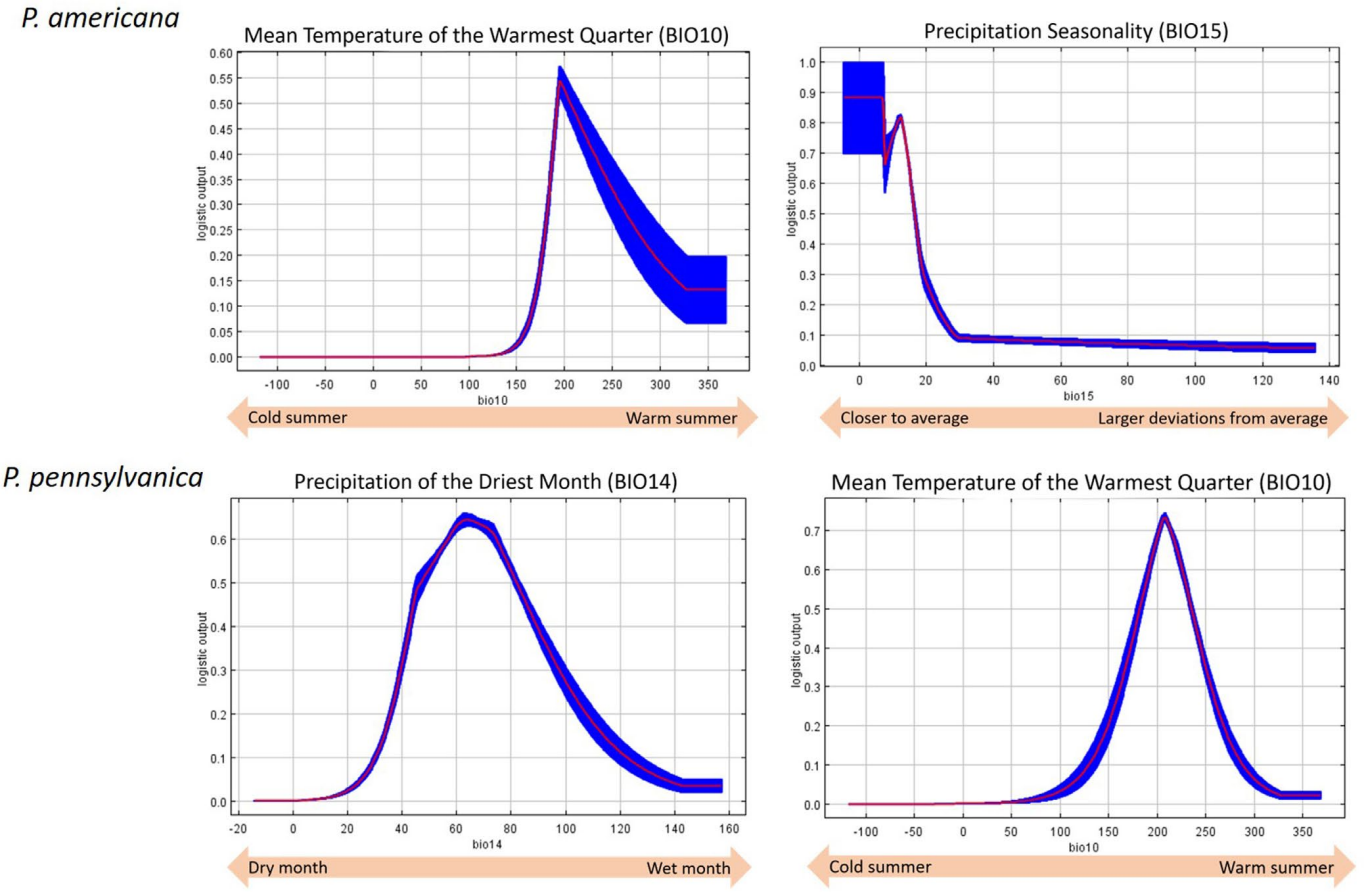
Response curves of *P. americana* (top) and *P. pennsylvanica* (bottom) to their strongest respective predictors. Red indicates the mean response averaged over the 10 replicate Maxent runs, while blue indicates one standard deviation. For *P. americana*, BIO10 (Mean Temperature of the Warmest Quarter) had the largest percent contribution and the largest permutation importance, followed by BIO15 (Precipitation Seasonality). For *P. pennsylvanica*, BIO14 (Precipitation of the Driest Month) had the largest percent contribution, and BIO10 (Mean Temperature of the Warmest Quarter) had the largest permutation importance

For both species, precipitation and temperature were identified as the strongest predictor of occurrence (see Appendix S2 for the contributions of isolated predictors on models). Mean Temperature of the Warmest Quarter (BIO10) was the environmental variable with the largest relative percent contribution to the *P. americana* ranges. Response curves indicate that the highest probability of *P. americana* occurrence was at an average temperature of about 19°C during the warmest quarter. This was also the environmental variable with the greatest permutation importance. A second bioclimatic predictor that contributed strongly to the *P. americana* model is Precipitation Seasonality (BIO15), the deviation of monthly precipitation from the annual average. Response curves indicate that the highest probability of *P. americana* occurrence is also dependent on a lower precipitation seasonality, that is, lower monthly precipitation variation. For *P. pennsylvanica*, precipitation was implicated as the most important factor as indicated in the response curves (Figure 3), namely Precipitation of the Driest Month (BIO14). *P. pennsylvanica* has the highest probability of occurrence below precipitation levels of about 40 millimeters; above precipitation levels of 100 millimeters, the probability of *P. pennsylvanica* occurrence decreases to less than half of the probability of occurrences at optimal precipitation. Additionally, there was again support for the Mean Temperature of the Warmest Quarter (BIO10) as it was the variable with the greatest permutation importance. The probability of *P. pennsylvanica* occurrences was greatest at a temperature of about 21°C during the warmest quarter. Above a temperature of 23°C and below a temperature of 18°C, the probability of *P. pennsylvanica* occurrences decrease to less than half of the probability of occurrences at optimal temperatures. Jacknife plots using testing data highlight the relative importance of variables that contributed to the model (Figure S2.3, Appendix S2).

**FIGURE 4:**
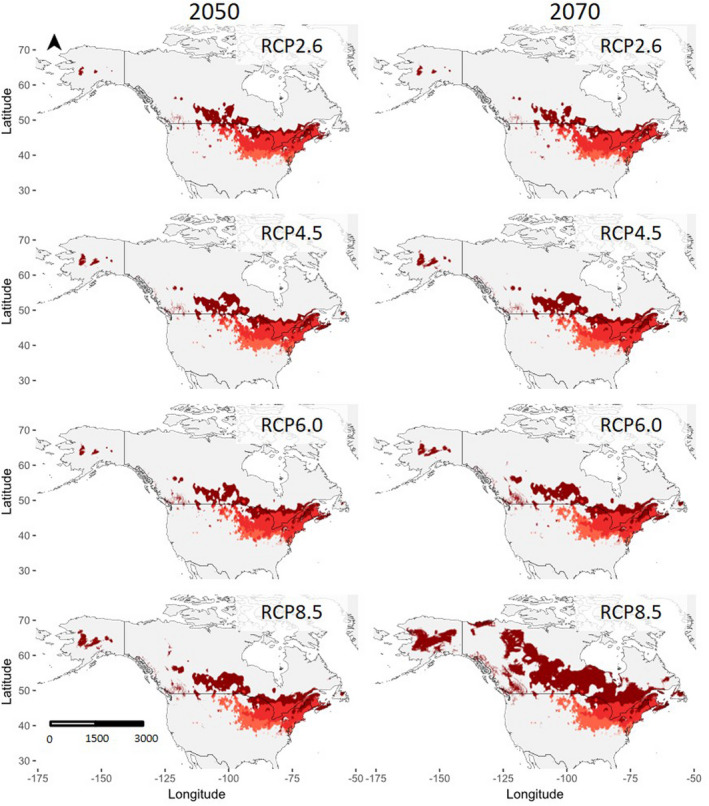
Projected future distributions of *P. americana* in 2050 (left) and 2070 (right), relative to current distributions. From top to bottom, the modeled projections show distributions in RCP2.6, RCP4.5, RCP6.0, and RCP8.5. Dark red indicates previously unsuitable habitats that have become suitable, while light red indicates previously suitable habitats that have become unsuitable

**FIGURE 5:**
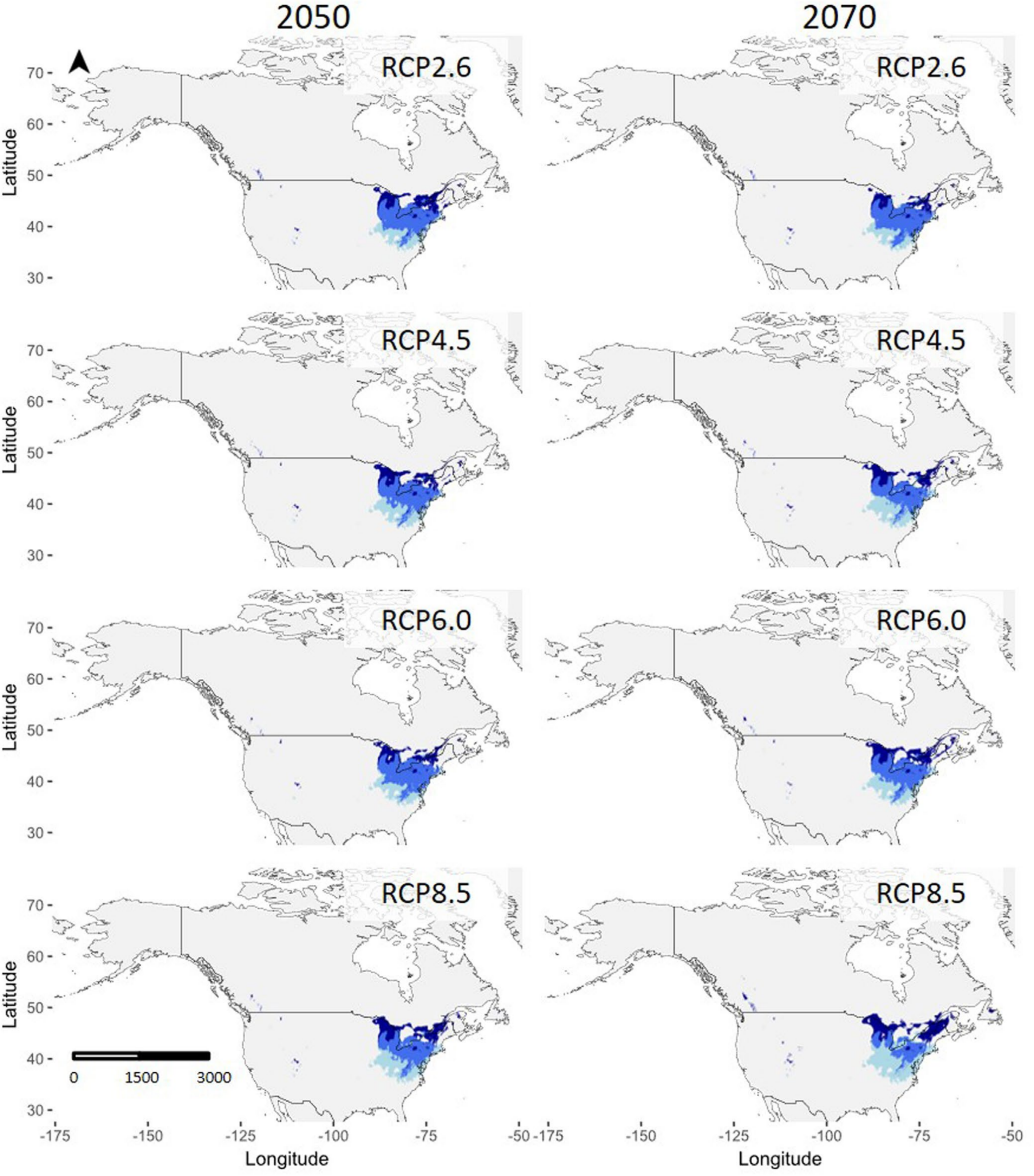
Projected future distributions of *P. pennsylvanica* in 2050 (left) and 2070 (right), relative to current distributions. From top to bottom, the modeled projections show distributions in RCP2.6, RCP4.5, RCP6.0, and RCP8.5. Dark blue indicates previously unsuitable habitats that have become suitable, while light blue indicates previously suitable habitats that have become unsuitable

Predicted future distributions were mapped as habitat suitability models for *P. americana* (Figure 4) and *P. pennsylvanica* (Figure 5), and the predicted changes in suitable habitats are summarized as percentages (Table 1). At all RCP projections, the percentage of suitable habitats is predicted to increase for *P. americana*, with larger increases predicted for scenarios of higher greenhouse gas emissions. The greatest percent increase of suitable habitats occurs at RCP8.5, with a 4.2% increase in 2050 and a 14.7% increase in 2070, compared to current suitable habitats. The direction of the range increase is largely northwestward. The percentage of suitable habitats for *P. pennsylvanica* stays constant or decreases when projected for 2050 and 2070. The smallest change occurs at RCP2.6, in which *P. pennsylvanica* ranges are predicted to only decrease by 0.2% in 2070. Conversely, the greatest percent decrease of suitable habitats for *P. pennsylvanica* occurs at RCP8.5, at which ranges will shrink by 0.3% in 2050 and 0.6% in 2070. The predicted range contractions occur largely in the southern portion of current ranges, while range expansions are northward. Notably, the change in the percentage of suitable habitats is very small (all less than 1%). However, there are some fluctuations around range edges where suitable habitat is expected to become unsuitable and vice versa. The greatest decreases of suitable relative to current suitable habitats occur at RCP4.5 and RCP8.5.

**TABLE 1:** The percentage of change of suitable habitat under different representative concentration pathway (RCP) trajectories in comparison with current predicted distributions

	Year	RCP2.6	RCP4.5	RCP6.0	RCP8.5
*P. americana*	2050	+2.7%	+2.8%	+3.4%	+4.2%
	2070	+2.3%	+3.5%	+3.9%	+14.7%
*P. pennsylvanica*	2050	+0.0%	−0.3%	−0.2	−0.3%
	2070	−0.2%	−0.5%	−0.1%	−0.6%

**FIGURE 6:**
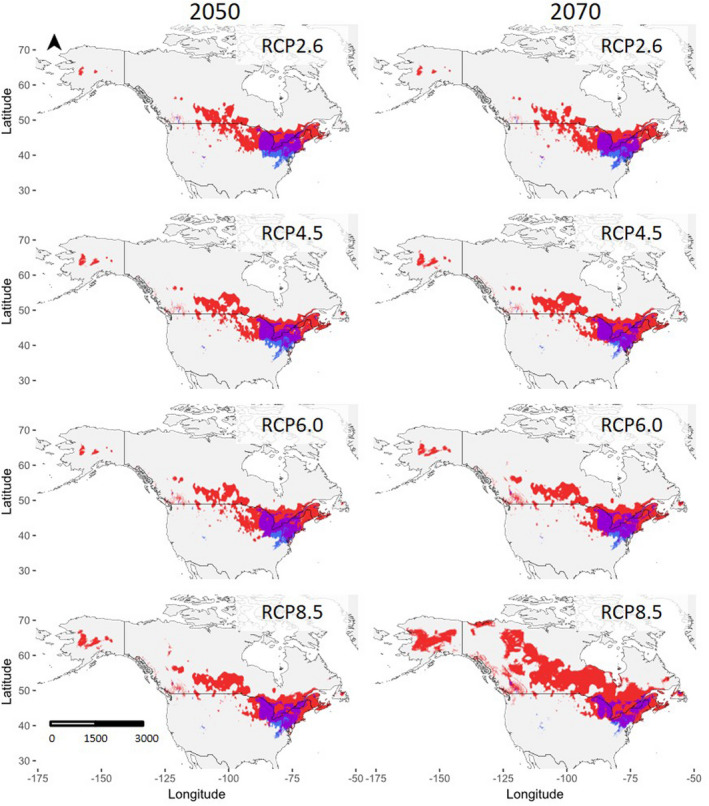
Projected distributions and overlap of *P. americana* and *P. pennsylvanica* in 2050 (left) and 2070 (right). From top to bottom, the modeled projections show distributions in RCP2.6, RCP4.5, RCP6.0, and RCP8.5. Red indicates locations that are only suitable for *P. americana*, blue indicates locations that are only suitable for *P. pennsylvanica*, purple indicates locations that are suitable for both taxa, and gray indicates locations that are unsuitable for both taxa

At all RCP trajectories, there is a slight increase in overlapping ranges of the two species (Figure 6; also, Table S3.1 and S3.2, Appendix S3). The largest increase in overlap occurs at RCP6.0, when the overlap increases by 0.3% in 2050 and 0.5% in 2070. However, this increase in overlapping regions translates to a contraction of regions that contain only a single species. That is, there is a decrease in habitat suitable only for *P. pennsylvanica*, but habitats that currently only contain *P. americana* are predicted to increase. This suggests that the changes in the amount of suitable habitat will result in the range shift of *P. americana* toward the range of *P. pennsylvanica*, resulting in a larger degree of overlap. Additionally, at all RCP trajectories, the amount of unsuitable habitat decreases, with the greatest decrease of 3.6% in 2050 and 13.8% in 2070 occurring at RCP8.5; most of the previously unsuitable habitat is becoming only suitable for *P. americana*. Greater RCP trajectories result in greater decreases in the amount of unsuitable habitat.

## 4 DISCUSSION

Our models predict different responses of *P. americana* and *P. pennsylvanica* to anthropogenic climate change, which may correspond to their respective niche requirements. Our forecasts predict range expansions of *P. americana* into 2050 and 2070, while *P. pennsylvanica* ranges are expected to remain the same or contract (see Appendix S3). This suggests that the ranges of both species may be able to keep up with short‐term predicted climate change. However, by 2070, only *P. americana* ranges are predicted to experience rapid expansion. It should be noted that the occurrence and environmental data used in this study span 1970 to 2000, which we referred to as the “current” range. Naturally, validating the predictions derived from the models will require observation and updating as the movement (presumably) proceeds.

Species that inhabit the same geographic range may exhibit high ecological similarity, but imperfect niche overlap will permit coexistence (Darwell & Althoff, 2017). The distinct yet overlapping distributions of *P. americana* and *P. pennsylvanica* suggest that different bioclimatic variables act to limit ranges. Here, we identify the variables that are candidates for determining the ranges of *P. americana* and *P. pennsylvanica*.

Our analyses consistently implicate precipitation as an important determinant of the abiotic limits of both species, whereby *P. americana* and *P. pennsylvanica* have different optima. Our results highlight the possibility that natural selection mediated by abiotic factors may be specific to life stage (Arnold & Wade, 1984). For example, the driest month corresponds to the period when eggs of both species are dormant in winter diapause. During this period, eggs, which are laid on plant material at the end of summer, are likely to be near the ground, and the amount of precipitation might translate to potential vulnerability to flooding or inundation in the following spring. Previous work in one species (Mason, 1976) has also invoked winter conditions during the egg stage as an important period for triggering phenotypically plastic responses later in life. For many organisms, strong fluctuations in the environment can be the source of severe selection, possibly explaining why our analyses also recovered variability in precipitation (during both winter and summer, when juvenile and adult bugs are present) as important in predicting occurrences (i.e., exclusion).

Mirroring the results for precipitation, our analyses also identified mean and variability of temperature as potentially important determinants of geographic distribution, albeit with different effects on the two taxa. The distribution of *P. pennsylvanica* was particularly dependent upon a lower average temperature of the warmest quarter, which is demonstrated by range contractions at higher RCP projections. This possibly points to challenges in feeding, mating behavior, or reproduction. In contrast, while *P. americana* distributions are also dependent on the mean temperature of the warmest quarter, its range is predicted to expand at higher RCP projections. Although identification of specific mechanisms is beyond the scope of the present work, the importance of temperature and fluctuating environmental conditions is consistent with an extensive body of literature on thermal ecology in insects, including a series of studies demonstrating fluctuating and temperature‐dependent selection in ambush bugs (Punzalan et al., 2008a; Punzalan, Rodd, & Rowe, 2008b, 2010).

A sometimes underappreciated underlying assumptions of the models is that species are currently in equilibrium with the environment and that species ranges are expected to shift as a consequence of changing environmental conditions (Elith et al., 2010; Guisan & Thuiller, 2005). For example, despite our data suggesting that *P. pennsylvanica* has a relatively restricted range, it is possible that both *Phymata* species are still responding to a recent climatic event, or have already begun responding to recent climate change at the time that occurrence and climatic data were collected. The predicted distributions for *P. pennsylvanica* indicate particularly prominent variation around this species’ range edges, possibly indicating that *P. pennsylvanica* is near its climatic optimum (Araujo & Pearson, 2005; Hutchinson, 1957). In comparison, there appears to be more habitat that satisfies the niche of *P. americana* given different climate change scenarios. Although it seems contradictory, at first, that the range of suitable habitat for *P. pennsylvanica* is not predicted to shift northward in response to forecasted environmental conditions, we propose an explanation. As suitable habitats for *P. pennsylvanica* are predicted to be strongly dependent on winter precipitation levels and summer temperature, it is possible that these future conditions do not change as drastically as other environmental variables that have lesser effects, as predicted by the model.

Although it remains to be seen whether the forecasted changes in climatic conditions are realized, the predicted range expansions and potential range overlap suggest increased hybridization opportunities and a larger arena for competition. The potential consequences are difficult to predict and depend on a number of factors including rates of dispersal, the fitness of hybrids, and the possibility of character displacement (Goldberg & Lande, 2007; Pfennig et al., 2016). Such biotic interactions are not accounted for in our models, but will almost certainly have important influence on realized distributions (Hof et al., 2012). This also highlights a critical limitation of SDM in that they typically omit biotic interactions (Bulgarella et al., 2014), and the integration of biological interactions with abiotic information remains one of the frontiers in modeling species distributions (Anderson, 2017; Elith & Leathwick, 2009). Furthermore, the models only make projections of potentially suitable habitats, but do not exclude the possibility that some populations may successfully persist at or beyond the predicted range margins of the “preferred” habitat (e.g., due to local adaptation and/or metapopulation dynamics). There is also no guarantee that populations will always successfully track spatial shifts in environmental regimes, in which case the models may underestimate the possibility and rate of local extirpation. Nevertheless, our models provide a starting point for generating hypotheses regarding climate change effects on ambush bugs and add to a growing recognition that the current trajectory of climate warming can have important eco‐evolutionary ramifications.

Overall, our results are consistent with effects of climate change that is highly variable across species, geographic regions, and over time (Menzel et al., 2002). In other taxa, a diverse spectrum of range shifts has been well documented (Chen et al., 2011). Variability in responses to different climate change scenarios at different timepoints in the future is seen in studies that have investigated both individual species (Dowling, 2015; Ning et al., 2017) and groups of species (Rebelo et al., 2010; Urbani et al., 2017). Different emissions scenarios (i.e., different RCPs) may have opposite effects on distributions, where a lower RCP induces range expansions and higher RCP projections lead to range contractions (Wang et al., 2018). Temporal variation has also been reported, where species were predicted to face extinction due to climate change at the end of the century, even though current distributions were predicted to expand (Rebelo et al., 2010). Additionally, predicted trends of range shifts may also be dependent on the amount of uncertainty incorporated in climate data sets (Parra & Monahan, 2008). For instance, our present study used four climate change projections in order to capture several potential future distributions, but there are several other projected concentration pathways that encompass a wider range of possible future greenhouse gas emissions. Due to the variability in these predictions, modeled scenarios should be used as guides that are ultimately supplemented by additional sampling or modeling; any long‐term trends may be obscured by short‐term range expansions or contractions. The use of SDM such as Maxent is critical tools for predicting range shifts, but these distributions are contingent upon the emission scenarios used.

Errors in species occurrence data are virtually inevitable, resulting from inaccuracies in georeferencing, imprecision in latitude and longitude coordinates, spatial autocorrelation of occurrence data, or uncertainty in locality descriptions. However, relative to other species distribution modeling methods based on occurrence data, Maxent has been found to maintain predictive accuracy even with locational errors. Maxent is also less sensitive to a limited sample compared to other SDM (Wisz et al., 2008) and performs well, so long as the data are comprised of widely distributed localities. The data used in our models originated from multiple sources and databases and consisted of samples across much of the previously assumed range of *Phymata*, though the subset of data eventually retained was notably depauperate of *P. americana* from the southwestern United States. Although Maxent is known to perform well, based on AUC, even in the presence of spatial sampling bias (Fourcade et al., 2014), this does raise a concern about model accuracy that is universal to virtually all SDM, and the appropriate remedy for such bias is not clear. Nevertheless, our goal was to examine potential range shifts in regions pertinent to possible hybridization, and perhaps a cautious approach is to interpret our results as predictions confined to a subset of the geographical range. Citizen science data may be particularly prone to opportunistic collection, and hence, biased occurrence data (Syfert et al., 2013; Tiago et al., 2017). Conversely, citizen‐collected data have been found to complement systematic collections, and models from these different data sources are largely consistent (Henckel et al., 2020). In the present study, the contributions of citizen science data were limited to only three data points retained in any of the model; the subset of the observations that temporally overlapped with the available environmental data happened to be comprised mostly of museum data.

A potentially more pressing concern is the errors arising from species misidentification (i.e., misidentifying *P. pennsylvanica* individuals as *P. americana* or vice versa), as it may result in seemingly robust but inaccurate models (Lozier et al., 2009). It is for this reason that we attempted to remove data corresponding to questionable identifications, which included a large portion of the citizen science data. We stress the importance of validation by experts, as incorrect species identification can negatively affect the quality of citizen science records (Geldmann et al., 2016). Future studies involving species distribution modeling could surely benefit from the addition of citizen science data as these databases improve (Tiago et al., 2017) and provided that species occurrence data are accurate and sufficiently widely distributed.

## 5 CONCLUSION

This study provides evidence for specific environmental requirements for *P. americana* and *P. pennsylvanica*, and these variables contribute to our limited understanding of the realized niches of both ambush bug species. We identified temperature and precipitation as important predictors, although with different effects on the distributions of each species. Projections under various climate change scenarios generally suggest a more substantial range expansion of *P. americana* than for *P. pennsylvanica*. Our models also predicted an increase in overlap of respective ranges, suggesting increased opportunities for hybridization, and highlighting the potentially important role of anthropogenic effects on this process.

## CONFLICT OF INTEREST

The authors declare no conflicts of interest.

## AUTHOR CONTRIBUTIONS

**Vicki Mengyuan Zhang:** Conceptualization (equal); Formal analysis (equal); Investigation (lead); Methodology (lead); Writing‐original draft (lead); Writing‐review & editing (equal). **David Punzalan:** Conceptualization (equal); Data curation (lead); Formal analysis (equal); Investigation (supporting); Methodology (supporting); Supervision (equal); Writing‐original draft (supporting); Writing‐review & editing (equal). **Locke Rowe:** Funding acquisition (lead); Resources (lead); Supervision (equal); Writing‐review & editing (equal).

### OPEN RESEARCH BADGES

This article has earned an Open Data Badge for making publicly available the digitally‐shareable data necessary to reproduce the reported results. The data is available at https://doi.org/10.17605/OSF.IO/B5H3A.

## Supporting information

Appendix S1–S3

## Data Availability

The data used in this study have been uploaded to the Open Science Framework and can be accessed at the following https://doi.org/10.17605/OSF.IO/B5H3A.
