# Supplementary material for: Climate change has different predicted effects on the range shifts of two hybridizing ambush bug (Phymata, Family Reduviidae, Order Hemiptera) species
Source: Ecol Evol. 2020 Oct 15;10(21):12036–48. doi: 10.1002/ece3.6820 (PMC7664010; doi:10.1002/ece3.6820)
Supplement: Supplementary file 1 — Appendix S1–S3 [file ECE3-10-12036-s001.docx]

## **Appendix 1**


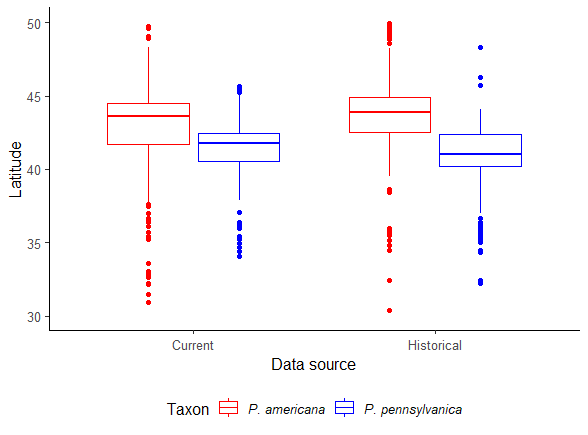


Figure S1.1. Current and historical distributions of P. americana in red and P. pennsylvanica in blue. Current observations include any observations from 1970 (inclusive) to present, while historical observations include any observations prior to 1970); this includes both museum and citizen science data and spans a time frame from 1864-2018. There is a total of 1075 P. americana sightings (414 current observations and 661 historical observations) and 970 P. pennsylvanica sightings (388 current observations and 582 historical observations). Comparison of distributions suggest an increase in overlapping ranges in current ranges.

Table S1.1. Summary of total observations used in MAXENT and their source.

| source | *P. americana* | *P. pennsylvanica* |
| --- | --- | --- |
| AMNH | 1 | 3 |
| Carnegie | 5 | 4 |
| CNC | 28 | 2 |
| iNat | NA | 3 |
| PBI | 20 | 82 |
| ROM | 30 | NA |
| UBC | 13 | NA |
| UMichigan | 5 | 26 |
| UofGuelph | 2 | 2 |
| Total | 104 | 122 |

Table S1.2. Bioclimatic variables and their respective code, scaling factor and units. The descriptions used were from the US Geological Survey (O’Donnell & Ignizio, 2012). All the variables were scaled by some factor, and this is reflected in the x-axis of the response curves. The variables used for Maxent models are indicated in bold.

| **Code** | **Variable** | **Description** | **Scaling Factor** | **Units** |
| --- | --- | --- | --- | --- |
| BIO1 | Annual Mean Temperature | The average temperature for each month | 10 | °C |
| **BIO2** | **Mean Diurnal Range** | **Mean of monthly difference between the maximum and minimum temperature** | **10** | **°C** |
| BIO3 | Isothermality | Mean diurnal range (BIO2) divided by temperature annual range (BIO7) multiplied by 100 | 100 | Dimension-less |
| BIO4 | Temperature Seasonality | The amount of temperature variation over  a given year based on the standard  deviation of monthly temperature averages. | 100 | °C |
| BIO5 | Max Temperature of Warmest Month | The maximum monthly temperature occurrence over a given year | 10 | °C |
| BIO6 | Min Temperature of Coldest Month | The minimum monthly temperature occurrence over a given year | 10 | °C |
| **BIO7** | **Temperature Annual Range** | **Difference between maximum temperature of warmest month and minimum temperature of coldest month (BIO5–BIO6)** | **10** | **°C** |
| **BIO8** | **Mean Temperature of Wettest Quarter** | **The average temperature of the wettest quarter** | **10** | **°C** |
| BIO9 | Mean Temperature of Driest Quarter | Average temperature of the driest quarter | 10 | °C |
| **BIO10** | **Mean Temperature of Warmest Quarter** | **Average temperature of the warmest quarter** | **10** | **°C** |
| BIO11 | Mean Temperature of Coldest Quarter | Average temperature of the coldest quarter | 10 | °C |
| BIO12 | Annual Precipitation | Sum of all total monthly  precipitation values. | 1 | Millimeters |
| BIO13 | Precipitation of Wettest Month | Total precipitation during the wettest month | 1 | Millimeters |
| **BIO14** | **Precipitation of Driest Month** | **Total precipitation during the driest month** | **1** | **Millimeters** |
| **BIO15** | **Precipitation Seasonality (Coefficient of Variation)** | **Variation in monthly precipitation; the standard deviation of the monthly total precipitation and the mean monthly total precipitation expressed as a ratio** | **100** | **Fraction** |
| BIO16 | Precipitation of Wettest Quarter | Total precipitation of the wettest quarter | 1 | Millimeters |
| BIO17 | Precipitation of Driest Quarter | Total precipitation of the driest quarter | 1 | Millimeters |
| **BIO18** | **Precipitation of Warmest Quarter** | **Total precipitation of the warmest quarter** | **1** | **Millimeters** |
| **BIO19** | **Precipitation of Coldest Quarter** | **Total precipitation of the coldest quarter** | **1** | **Millimeters** |

|  | BIO2 | BIO7 | BIO8 | BIO10 | BIO14 | BIO15 | BIO18 | BIO19 |
| --- | --- | --- | --- | --- | --- | --- | --- | --- |
| BIO2 | 1.000 |  |  |  |  |  |  |  |
| BIO7 | -0.058 | 1.000 |  |  |  |  |  |  |
| BIO8 | 0.658 | 0.047 | 1.000 |  |  |  |  |  |
| BIO10 | 0.822 | -0.252 | 0.811 | 1.000 |  |  |  |  |
| BIO14 | 0.066 | -0.477 | 0.114 | 0.342 | 1.000 |  |  |  |
| BIO15 | -0.038 | 0.267 | 0.051 | -0.179 | -0.748 | 1.000 |  |  |
| BIO18 | 0.236 | -0.284 | 0.470 | 0.488 | 0.760 | -0.449 | 1.000 |  |
| BIO19 | 0.103 | -0.600 | 0.006 | 0.312 | 0.766 | -0.454 | 0.525 | 1.000 |

Table S1.3. Collinear matrix for the 8 bioclimatic variables used in Maxent modeling under current (1970-2000) conditions.

|  | BIO2 | BIO7 | BIO8 | BIO10 | BIO14 | BIO15 | BIO18 | BIO19 |
| --- | --- | --- | --- | --- | --- | --- | --- | --- |
| BIO2 | 1 |  |  |  |  |  |  |  |
| BIO7 | 0.048663 | 1 |  |  |  |  |  |  |
| BIO8 | 0.64652 | 0.141092 | 1 |  |  |  |  |  |
| BIO10 | 0.845342 | -0.12033 | 0.777043 | 1 |  |  |  |  |
| BIO14 | 0.102094 | -0.40802 | 0.091936 | 0.343762 | 1 |  |  |  |
| BIO15 | -0.05848 | 0.145339 | 0.031789 | -0.18936 | -0.74809 | 1 |  |  |
| BIO18 | 0.203838 | -0.24716 | 0.395557 | 0.431682 | 0.792632 | -0.49361 | 1 |  |
| BIO19 | 0.132675 | -0.548 | -0.01958 | 0.313279 | 0.741629 | -0.42259 | 0.53189 | 1 |

Table S1.4. Collinear matrix for the 8 bioclimatic variables used in Maxent modeling under future conditions.

## **Appendix 2**

Table S2.1. Model performance evaluation in the form of the True Skill Statistic (TSS) and the Area Under the Curve (AUC) for *P. americana* and *P. pennsylvanica*. Values given are the average over 10-fold cross validation ± standard deviation.

|  | TSS | AUC |
| --- | --- | --- |
| AM | 0.75 ± 0.18 | 0.964 ± 0.028 |
| PE | 0.80 ± 0.14 | 0.980 ± 0.010 |

Table S2.2. Analysis of variable contribution for P. americana. Percent contributions indicate the increase in gain to the model from an environmental variable. Permutation importance indicates the significance of the predictor.

| **Variable** | **Percent contribution** | **Permutation importance** |
| --- | --- | --- |
| BIO10 | **39.5** | **76.5** |
| BIO15 | 37.7 | 0.8 |
| BIO2 | 8 | 15.6 |
| BIO14 | 6.7 | 0.1 |
| BIO7 | 4.3 | 6.1 |
| BIO18 | 2.8 | 0.2 |
| BIO19 | 0.9 | 0.7 |
| BIO8 | 0.1 | 0.1 |

| **Variable** | **Percent contribution** | **Permutation importance** |
| --- | --- | --- |
| BIO14 | **26.4** | 0.8 |
| BIO15 | 24.8 | 29.8 |
| BIO10 | 24.7 | **40** |
| BIO8 | 9.6 | 2 |
| BIO7 | 9.6 | 17.4 |
| BIO19 | 2.4 | 2.9 |
| BIO2 | 2.3 | 6.9 |
| BIO18 | 0.2 | 0.3 |

Table S2.3. Analysis of variable contribution for P. pennsylvanica. Percent contributions indicate the increase in gain to the model from an environmental variable. Permutation importance indicates the significance of the predictor.


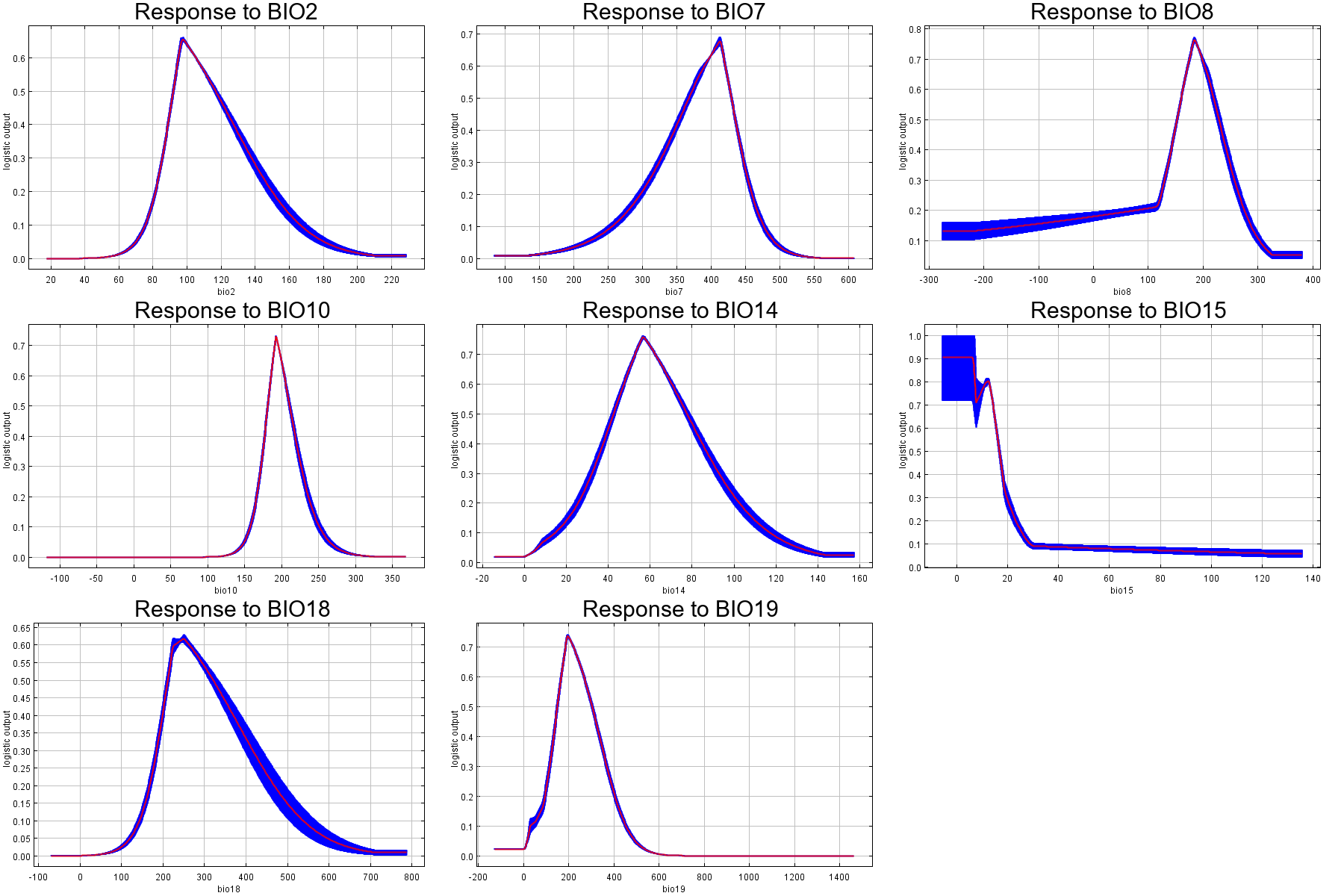


Figure S2.1. Response curves of P. americana to the 8 bioclimatic variables. Each curve is a different model using the variable listed in isolation (i.e., omitting the other 7 variables). Red indicates the mean response averaged over the 10 replicate MAXENT runs, while blue indicates one standard deviation.


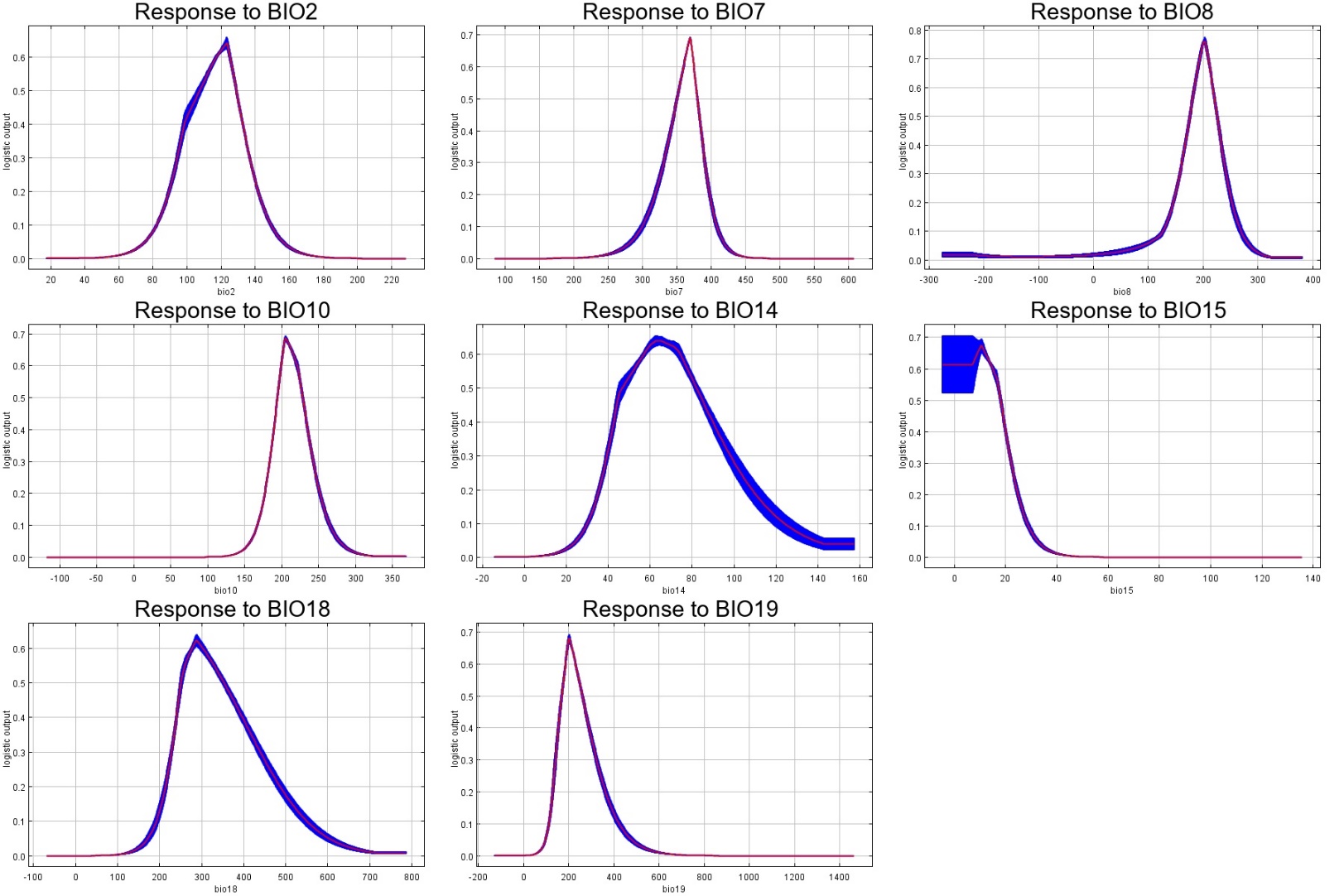


Figure S2.2. Response curves of P. pennsylvanica to the 8 bioclimatic variables. Each curve is a different model using the variable listed in isolation (i.e., omitting the other 7 variables). Red indicates the mean response averaged over the 10 replicate MAXENT runs, while blue indicates one standard deviation.


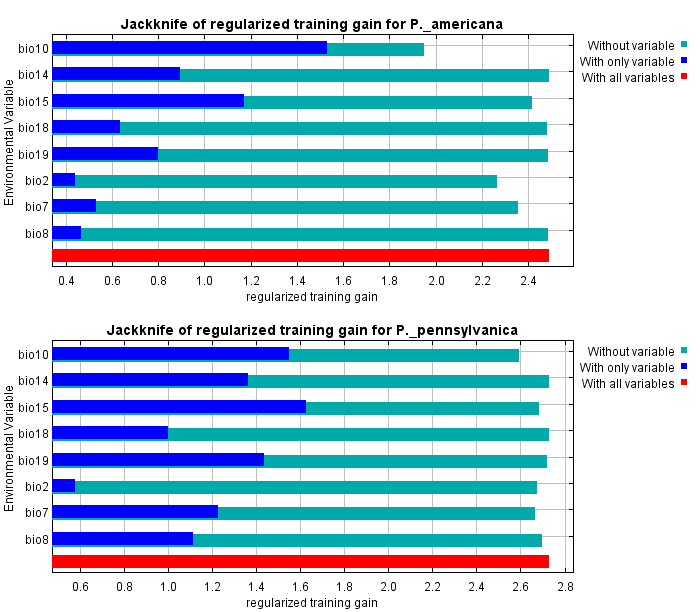


Figure S2.3. Jacknife plots for P. americana (top) and P. pennslvanica (bottom).

## **Appendix 3**

Table S3.1. The percentage, out of all potential habitats, of all suitable and unsuitable habitat in 2050 climate projections. These percentages of suitable habitat for P. americana and P. pennsylvanica are relative to the total projected area, compared to current predicted suitable habitat.

| **RCP (2050)** | ***P. americana* only** | ***P. pennsylvanica* only** | **Overlap** | **No suitable habitat** |
| --- | --- | --- | --- | --- |
| 2.6 | + 2.3% | – 0.2% | + 0.2% | – 2.2% |
| 4.5 | + 2.8% | – 0.3% | 0 | – 2.5% |
| 6.0 | + 3.1% | – 0.5% | + 0.3% | – 2.9% |
| 8.5 | + 3.8% | – 0.6% | + 0.4% | – 3.6% |
|  |  |  |  |  |

Table S3.2. The percentage, out of all potential habitats, of all suitable and unsuitable habitat in 2070 climate projections. These percentages of suitable habitat for P. americana and P. pennsylvanica are relative to the total projected area, compared to current predicted suitable habitat.

| **RCP (2070)** | ***P. americana* only** | ***P. pennsylvanica* only** | **Overlap** | **No suitable habitat** |
| --- | --- | --- | --- | --- |
| 2.6 | + 2.3% | – 0.3% | 0 | – 2.1% |
| 4.5 | + 32% | – 0.7% | + 0.3% | – 2.8% |
| 6.0 | + 3.3% | – 0.6% | + 0.5% | – 3.3% |
| 8.5 | + 14.4% | – 0.9% | + 0.3% | – 13.8% |
